# Opioid-related harms and care impacts of conventional and AI-based prescription management strategies: insights from leveraging agent-based modeling and machine learning

**DOI:** 10.3389/fdgth.2023.1174845

**Published:** 2023-06-20

**Authors:** Narjes Shojaati, Nathaniel D. Osgood

**Affiliations:** Department of Computer Science, University of Saskatchewan, Saskatoon, SK, Canada

**Keywords:** agent-based modeling, machine learning, hidden Markov model, medical prescription for opioids, opioid-related harm

## Abstract

**Introduction:**

Like its counterpart to the south, Canada ranks among the top five countries with the highest rates of opioid prescriptions. With many suffering from opioid use disorder first having encountered opioids *via* prescription routes, practitioners and health systems have an enduring need to identify and effectively respond to the problematic use of opioid prescription. There are strong challenges to successfully addressing this need: importantly, the patterns of prescription fulfillment that signal opioid abuse can be subtle and difficult to recognize, and overzealous enforcement can deprive those with legitimate pain management needs the appropriate care. Moreover, injudicious responses risk shifting those suffering from early-stage abuse of prescribed opioids to illicitly sourced street alternatives, whose varying dosage, availability, and the risk of adulteration can pose grave health risks.

**Methods:**

This study employs a dynamic modeling and simulation to evaluate the effectiveness of prescription regimes employing machine learning monitoring programs to identify the patients who are at risk of opioid abuse while being treated with prescribed opioids. To this end, an agent-based model was developed and implemented to examine the effect of reduced prescribing and prescription drug monitoring programs on overdose and escalation to street opioids among patients, and on the legitimacy of fulfillments of opioid prescriptions over a 5-year time horizon. A study released by the Canadian Institute for Health Information was used to estimate the parameter values and assist in the validation of the existing agent-based model.

**Results and discussion:**

The model estimates that lowering the prescription doses exerted the most favorable impact on the outcomes of interest over 5 years with a minimum burden on patients with a legitimate need for pharmaceutical opioids. The accurate conclusion about the impact of public health interventions requires a comprehensive set of outcomes to test their multi-dimensional effects, as utilized in this research. Finally, combining machine learning and agent-based modeling can provide significant advantages, particularly when using the latter to gain insights into the long-term effects and dynamic circumstances of the former.

## 1. Introduction

The risks associated with psychoactive prescription medicines—particularly opioids—are significant issues of public health and patient safety (1–4), and Canada is still among the top five countries with the highest rate of opioid prescriptions for the last 15 years (5, 6). Some patients who were prescribed with opioids have sometimes resorted to non-medical opioid use or illicit drug supply due to various factors such as inadequate pain management, lack of access to alternative treatments, mental health issues, and addiction vulnerability (7, 8). In 2017, 11 lives were lost each day owing to opioid overdoses in Canada, with 3,658 opioid-related deaths reported in 2019 (9, 10). During the COVID-19 pandemic, this problem has become particularly acute, with sharp rises in death rates of opioid overdose (10, 11) and destabilizing effects in prescription opioids (12). Undeniably, the opioid crisis continues unmitigated in Canada (13); therefore, ensuring the safety of opioid use and adequate access to pain management should be among the top priorities for the healthcare system.

In recent years, a variety of policy interventions have been suggested to restrain medical opioid dispensing (14, 15); however, there are discrepant developments of decreasing opioid availability and increasing opioid mortality (16–18). Contradictions inherent in these interventions call for a systems science approach (19) to consider broader structural conditions contributing to the issue.

Systems science offers more holistic tools and framework for improving the understanding and decision-making regarding complex problems. Systems science methods enhance the capacity to reason about complex system behavior in systems marked by entangling of factors, feedbacks, path-dependence, delays and non-linearities, local contextual dependence, and distinct emergent behavior at different scales (20)—characteristics that are each notable features of the opioid crisis. Dynamic modeling within systems science supports alternative mechanisms for characterizing the structure of complex systems, which can aid in identifying key drivers that contribute to the emergence and persistence of complex phenomena, such as opioid use disorder (21, 22). Systems science and the dynamic simulation models can be used to explore the complex nature of the opioid crisis and study the effect of changes to the system with minimal costs, risks, and time (23).

A set of literature reviews (24–27) summarizes present existing research on the implemented dynamic models for prescription opioid use and harms. While dynamic modeling and simulation have been employed to study the different aspects of medical opioid dispensing, they have not taken into consideration the effectiveness of prescription regimes at the individual level, in general, or in the specific Canadian context that forms the focus of this work.

An agent-based model methodology can readily capture population heterogeneity and facilitate the study of a wide variety of the individual-level factors and their contribution to whole system behavior by simulating nature's evolution in opioid-prescribing practices, based on a set of specified rules. In comparison with previous dynamic modeling approaches that focus at an aggregate level, the individual-level characterization that is the hallmark of agent-based modeling supports capturing the effects of pro-social companionship and adverse social networks, feedback, and history dependence at an individual level—such as those associated with the development of tolerance and escalation of dosage levels and ensuring physiological dependence, stigmatization and the impact of adverse childhood experiences—widespread heterogeneity, as well as the effects of local context on a situated agent. With the application of an agent-based modeling, a new insight from a complex interaction of a whole system will often emerge, which was not seen before (28–30). Within the prescription context of the opioid use and misuse examined here, the utilization of an agent-based model allows the computer to evaluate different scenario results regarding altering prescribing practices, which can be examined and optimized with lower resources and cost than would be required for human trials (31). This paves the way for more exploratory use of the model as the source of the observed data and incorporation of a hidden Markov model (HMM) as a simulation model enhancement to implement a prescription drug monitoring program (PDMP) (32). A simple PDMP designed to prevent diversion and misuse of controlled substances by identification of possible “doctor–pharmacy shoppers” patterns (i.e., overlapping opioid prescriptions or obtaining multiple prescriptions from different prescribers and pharmacies) (33). However, an HMM-aided PDMP aims to monitor the legitimacy of prescriptions by leveraging the agent-based modeling and machine learning algorithms to identify potential cases of opioid overuse among patients.

A hidden Markov model (34) as a machine learning method has been used to capture hidden information from a sequence of observations over time. While there are various probabilistic sequence classification methods available (35–37), the HMM is specifically selected for this study over other approaches due to its strengths in handling sequential data, capturing hidden dynamics underlying the observed data, representing discrete hidden states, and modeling transitions between such hidden states. Furthermore, the HMM's solid mathematical foundation, satisfactory computational performance, and ability to provide a clear representation of the underlying model enable more meaningful interpretation of outcomes, particularly in the context of health policy studies (38, 39).

To employ an HMM, acquiring intensive longitudinal data is of utmost importance (34). A detailed set of data is utilized for estimating the probability of a sequence of observations, decoding the most likely sequence of hidden states underlying such a sequence, and training the HMM parameters based on those observations. An appropriate agent-based modeling framework has the potential to generate high-quality data that are specific for these purposes (40, 41). In this study, an HMM-aided PDMP is implemented in the agent-based model (42) to investigate whether the HMM could improve prescription drug monitoring programs to detect these unobserved legitimate states for any new opioid prescription filled by each patient and further study the consequence of the HMM-aided PDMP intervention.

The goal of the study is to emphasize the significance of utilizing dynamic modeling and simulation techniques with the integration of machine learning algorithms to investigate opioid use disorder among patients who have been prescribed with opioids. Utilizing an agent-based model is essential to serve as a basis for an HMM-aided PDMP and explore possible interventions while assessing any unforeseen consequences of different prescription regimes. Accordingly, this paper consists of the following sections: Section 2 presents the research methodology and provides an overview of the conceptual model, the agent-based model details, and the HMM-aided PDMP implementation and outlines different policy interventions; Section 3 discusses the different results of policy interventions; and Section 4 conveys the overall conclusions of this study along with policy suggestions.

## 2. Research methodology

In considering the complex nature of the opioid crisis (43, 44) and data gaps in this area (45, 46), it is evident that a conceptual model can facilitate developing an accurate and useful computational simulation model to support decision-making in the context of opioid therapy in the healthcare system (47).

### 2.1. Conceptual model for the opioid therapy agent-based model

The opioid crisis remains a significant public health challenge in Canada (13). Canada has one of the highest per capita consumptions of prescription opioids worldwide (48, 49). Prescription opioids are of particular concern, due to the potential harms associated with them, such as overdose and addiction (50). An estimated 8%–12% of patients who were prescribed with these medications developed dependence and started obtaining additional prescription opioids from different means such as overlapping opioid prescriptions, feigning symptoms of pain, or borrowing from other patients (7, 8). The quest to use increasing quantities may shift patients to street opioids (51). Potential shifts in opioid utilization and provision may lead to a move toward stronger illicit resources, beginning with heroin and possibly escalating to fentanyl (52, 53), to the extent that the prevalence of synthetic opioids like fentanyl in street opioids is responsible for 80% of all opioid-related deaths recorded in Canada in 2021 (10).

The nature of opioid use and outcomes includes many entangled components, rendering evaluation of the public health impact of any changes in opioid-prescribed practicing is extremely challenging. Particularly notable are challenges associated with balancing the provision of pain relief for those with acute chronic or transient pain, the desire to minimize the development of high levels of tolerance and physical dependency among those on prescription opioids, and the need to prevent individuals whose dosing is restrained in this way from switching to or supplementing their use with illegitimate requests for prescription or street-supplied opioids (54, 55).

**Figure 1:**
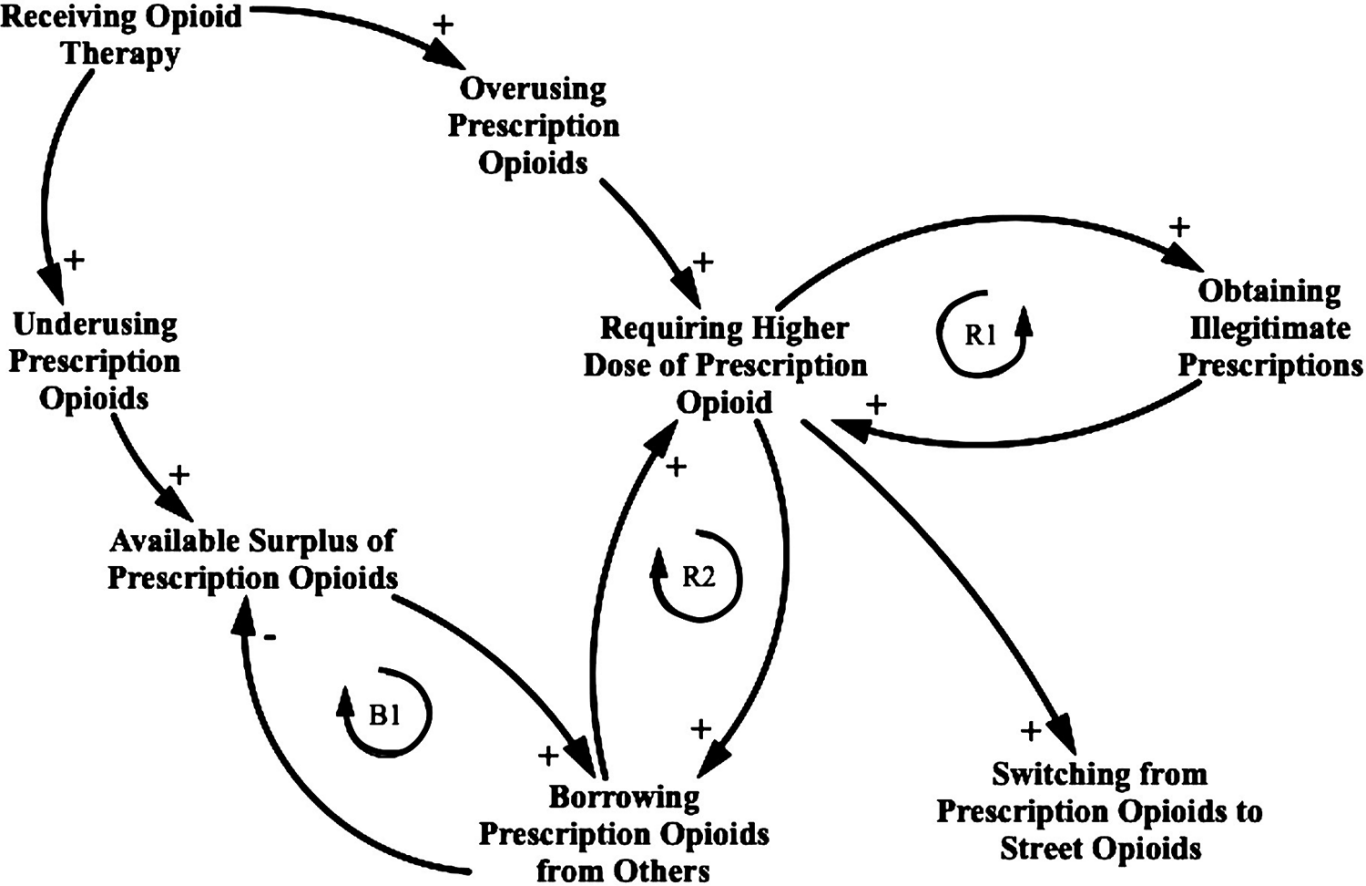
Causal loop diagram for the opioid prescription practicing consequence. This causal loop diagram consists of variables connected by arrows showing causal influence, with each relationship being positive (e.g., an increase in “Receiving opioid therapy” leads to an increase in “Overusing prescription opioids” compared with the value it otherwise would have held, *ceteris paribus*) or negative (e.g., an increase in “Borrowing prescription opioids from others” leads to a decrease in “Available surplus prescription opioid” compared with the value it otherwise would have held). Closed loops denote feedback, which is either reinforcing (R1 and R2) or balancing (B1). A switch to a supply of street opioids represents a situation where prescription-related factors may no longer apply.

Having stated the foregoing, Figure 1 represents a causal loop diagram for the opioid therapy agent-based model. This causal loop diagram shows the boundary of the model in terms of its breadth and highlights two reinforcing feedback loops and one balancing feedback loop in the current scope of the study. The researchers benefitted from published literature, and expert opinion in the development of this framework and consistent with the understanding gained through interaction with those with lived experience in this area.

As illustrated in Figure 1, the causal loop diagram assumes that a proportion of the population is misusing their prescription after the loss of adherence to opioid therapy, either by underusing or overusing opioids. A fraction of these people who start underusing might keep a portion of surplus opioids in their medication cabinet, ready to share with others. Another fraction of these people who start overusing might experience drug tolerance, which means that the dose must be increased over time to achieve the same effect (56). Therefore, they might engage in a few common mechanisms to obtain illegitimate prescriptions such as requesting frequent refills of opioid prescriptions, feigning symptoms of pain, forgery of prescription, and fraudulent telephone calls to pharmacies. These mechanisms are implemented in the model through the inclusion of either at least 1 day of overlapping opioid prescriptions or the procurement of new opioid prescriptions illicitly. The process creates the first reinforcing loop. The overusing patients who do not attempt to obtain illegitimate prescriptions seek surplus prescriptions acquired from others to address their increasing required doses. The process creates the second reinforcing loop. As the patients demand surplus opioid prescriptions, the overall source of surplus opioid prescriptions decreases. Because of this balancing feedback loop, the patients might shift to street opioids to address their needs.

Furthermore, the following statements describe the boundary of the model in terms of the depth of detail that was implemented for each person within the scope of the model. The model includes a population of 50,000 individuals, each characterized by two properties: opioid prescription dose and duration of treatment. Each individual has one type of social connection network, and the possible final states for an individual are either shifting to street opioid use or showing the signs of overdose.

### 2.2. Agent-based model and simulation

Designing a detailed conceptual model paved the way for developing and implementing a more detailed computational simulation model. As a result, an agent-based model was developed and implemented to examine the potential impact of reducing the opioid prescription dose and treatment durations, a simple PDMP and an HMM-aided PDMP on potentially important outcomes of interest such as medical and non-medical overdose, individuals who escalate to street opioids, legitimate, illegitimate, and total filled opioid prescriptions over a 5-year time horizon, which spans from 2013 to 2018. This time interval is consistent with the time period reported in the literature and does not include any temporary alterations in the opioid prescription practice that may have occurred during the COVID-19 pandemic. The agent-based model was developed using the simulation software AnyLogic version 8.8.1 (57), which is a simulation tool that performs the major types of systems science simulations (58). The model time unit is 1 day, and the model operates in a continuous time. The model is initialized after a 5-year burn-in period. After this 5-year burn-in period, the patients exhibit different histories states of use of prescribed opioids and resulting differences in adherence to opioid treatment. Therefore, the model started to show a meaningful and constant pattern after a 5-year burn-in period, and any calibration or experimentation is run after that period.

#### 2.2.1. Agent-based model structure and agent-characteristics

The agent-based model presented in this study characterizes the dynamics of opioid prescribing in Canada and can be used to evaluate different intervention responses to reduce harms associated with prescription opioid use. There is only one type of agent in this model: person. The dynamic of opioid prescribing for each person is captured *via* two state charts: the opioid-prescribing state chart (depicted in Supplementary Figure S1) and the medication adherence state chart (depicted in Supplementary Figure S2).

At the topmost level, the opioid-prescribing state chart (depicted in Supplementary Figure S1) characterizes whether the individual was or was not in opioid therapy in the past year. In the opioid therapy state, based on the duration of the prescription of the patient, that patient is further characterized as to whether they are on short-term therapy or long-term therapy. The people in the not in opioid therapy state transition into opioid therapy based on two different prescription initiation rates specified for new or established patients, respectively. These rates were determined based on the calibration experiment. To enter the opioid therapy state, firstly, the patients seek their prescriptions in one eponymous state and, upon receiving such prescriptions, move to the opioid therapy state. Another thing that occurs in the seeking prescription state is the HMM-aided PDMP, which classifies the requests for an opioid prescription to legitimate or illegitimate ones and therefore stops the person with an illegitimate opioid prescription from entering the opioid therapy state. Further information about the implantation of the HMM-aided PDMP is provided in Section 2.3.

As the patients initiate opioid therapy, a new opioid prescription dose and a new anticipated treatment length will be assigned to each patient based on custom distributions that are parameterized from CIHI data (48). Absent developing opioid overuse, all patients exit opioid therapy following the length of treatment based on timeout transitions.

Losing adherence to opioid treatment is implemented using the medication adherence state chart (depicted in Supplementary Figure S2), where people are divided into two groups *via* a binary representation of adherence to opioid treatment. When the patients receive an opioid prescription, they move to an adherence state. An internal timeout transition in adherence state calculates the remaining total prescription opioid dosage in case of loss of adherence. As time passes, each person might lose adherence based on a geometric growth function implemented inside an external timeout transition and move to a non-adherence state. Then the patients are divided into two main groups in non-adherence state, which are the underuse prescribed opioids or overuse prescribed opioids. This division is based on a possibility portion driven from literature. In the underuse state, each patient decides to store a portion of surplus prescribed opioids or dispose of them. After the initial treatment length finishes for the person in the underuse state, a transition fires, and the patients exit the underuse state and enter the free opioid state.

If a patient enters the overuse state after losing adherence to opioid treatment, the patient requires higher doses compared with the initial prescribed dose, which is calculated using a geometric growth function implemented within an internal timeout transition. Another internal timeout transition calculates the remaining opioid dose, and as the current available opioid prescription finishes, a transition fires; therefore, the patients start looking for other resources of available opioid prescription.

A portion of patients start to look for the possibility of obtaining illegitimate prescriptions. If any of the mechanisms for obtaining illegitimate prescriptions is successful, a transition fires and the patients move to the taking prescription opioids state. Some patients are not successful to obtain illegitimate prescriptions, as well as other patients who do not try obtaining illegitimate prescriptions start to investigate their network for others with surplus prescription opioids implemented inside a timeout transition. If a patient obtains prescription opioids from others that meet the needs of the patient in terms of the required opioid doses, a transition fires and moves the patient to the taking prescription opioids state.

Any successful obtaining of prescription opioids keeps the patients in the overuse state while using prescription opioids. The patients who fail to obtain prescription opioids move to the seeking street opioids state. A small portion of patients in the overuse state might stop using opioids and seek out of this state because of self-caring or treatment, based on a rate transition.

Patients at any state of opioid therapy have a transition to an overdose state governed by a hazard-rate based on the annual overdose rate considering recent opioid dose. The cumulative count of medical overdoses is calculated as the cumulative count of overdoses that occurred for the patients in the adherence state. The cumulative count of non-medical overdoses is calculated as the cumulative count of overdoses that occurred for the patients in the non-adherence state, which implies that the patients take a dosage other than the medically recommended one. (Note that other assumptions in translation from the real world into the opioid therapy agent-based model are provided in the Supplementary Table S2).

#### 2.2.2. Population and network

Agents were placed into a modeled environment of 50,000 persons, and the model incorporated a cohort population. The interaction of people in the model is essential when the patients start to look for surplus opioids through their connections. These interactions make use of a social network; for this purpose, the population was associated with an Erdos–Renyi network (59). Thus, people are connected randomly with a given average number of connections per person. Within the model, 60% of individuals know five or more people that aligns with the existing literature (60, 61) (see Supplementary Figure S3). The network connections for each individual are set at the initialization of the model and remain invariant throughout the simulation.

#### 2.2.3. Parameterization

A study released by the Canadian Institute for Health Information (CIHI) (48) provided the custom distribution of the prescribed dose and duration for new and established patient prescribed opioids within Canada. This study was used, together with information from a review of the literature on adherence with opioid therapy to estimate the parameter values needed for building the agent-based model. The information to estimate the occurrence of other relevant behaviors such as opioid overuse and overdose possibility was obtained from relevant literature, which the reported data should be adjusted for smoking, depression, pain site, age, and gender. Supplementary Table S1 summarizes the parameters and their sources.

#### 2.2.4. Sensitivity analyses, calibration, and validation of the model

Sensitivity analyses were conducted to identify the sensitive parameters in which the model outcome was affected by changes in them. Thus, the calibration was performed on these sensitive parameters to find their values that best replicated the reported data in the literature. During the calibration process, we manually varied a set of model parameters until the model outputs approximated the empirical data (see Supplementary Tables S3, S4). Furthermore, seven outcomes were utilized to validate the outcomes of the agent-based model over a 5-year time horizon against the empirical data reported by CIHI (48) or in the related literature including the proportion of people starting opioids without being prescribed with opioids in the past year and the proportion of patients on opioid therapy (see Supplementary Figure S4), the proportion of patients prescribed with opioids who either underuse or overuse them, and the proportion of patients who develop an opioid use disorder after being prescribed with opioids and subsequently overuse them (see Supplementary Figure S5), the proportion of patients on long-term opioid therapy and the proportion of patients who overuse prescription opioids transition to street opioids (see Supplementary Figure S6) and the prevalence of illegitimate opioid prescriptions (see Supplementary Figure S7).

At that point, a set of requirements to establish the model's credibility and validity was met, and the model is deemed suitable as a source of observed data to train the HMM-aided PDMP. It also explores different policies targeting the reduction of prescription opioid misuse.

### 2.3. Prescription drug monitoring program implementation using the two-state hidden Markov model

One of the most challenging tasks for any practitioner is to assess the legitimacy^1^ of prescriptions (63) for controlled substances (64). In the implemented model, as different studies of the prescription opioid use have found (65–67), some patients are more likely to seek additional prescription opioids due to building tolerance. They may try to fulfill their need through different pathways such as obtaining extra opioid prescriptions (68). In this case, the prescription opioids are no longer safe and effective in treating the medical condition of the patient and, therefore, potentially used for illegitimate purposes. Moreover, there is always some chance for these people to stop seeking additional prescription opioids based on self-care or treatment (69). Therefore, considering the ultimate goal of a prescription drug monitoring program to facilitate the fulfillment of legitimate prescriptions while preventing illegitimate ones, it is justifiable to incorporate two unobserved states, namely, “Legitimate” and “Illegitimate,” for each new opioid prescription, which rely on the present state of adherence of the patient and are not directly observable to practitioners.

Due to the challenges in obtaining integrated longitudinal data of the patients undergoing opioid prescription treatment, the training data for the HMM was produced by running the above-calibrated agent-based Monte Carlo simulation model with 10 realizations (each equipped with a different random seed) for 10 years. Consequently, the data for a subset of agents were collected to serve as the training data set for the HMM. By employing random sampling techniques, the Monte Carlo simulation allows for the exploration of a wide range of possible scenarios and outcomes, facilitating a broad understanding of the behavior of the system. The Monte Carlo simulation involved ensembles of 10 realizations, each possessing a unique random seed, to help capture the inherent variability in the system and enhance the robustness of the analysis. The conceptual model assumes that the patients initially adhere to opioid prescriptions. Therefore, the model is simulated for a duration of 10 years, and the data from the entire period for each individual is retained to maintain the desired initial probability of starting from an adherence state, regardless of the fact that the HMM-aided PDMP can be utilized at any point in the opioid therapy trajectory of a patient. Therefore, by utilizing a Monte Carlo simulation with 10 realizations over a 10-year period and drawing upon the data from a limited subset of agents, a robust training data set was generated for the HMM-aided PDMP. The validation data set, on the other hand, was obtained by excluding the initial 5-year burn-in period and encompassing a considerable population of agents. In addition, a Monte Carlo simulation with 100 realizations was conducted, introducing novel and previously unseen data for validation purposes. This approach facilitates the evaluation of the performance of the HMM-aided PDMP and its capacity to extend beyond the confines of the training data, thereby enhancing its reliability and effectiveness. To explore the receiver operating characteristic (ROC) curve, the HMM threshold range was set between 0 and 1, with an incremental step of 0.1. However, when there is a significant jump between outcomes in the receiver operating characteristic and precision-recall curves, the incremental step was reduced to 0.01. This range allows for a comprehensive exploration of different threshold values and their impact on the classification of prescriptions as legitimate or illegitimate opioid prescriptions.

Throughout each such realization, the data for any new opioid prescription claimed by each patient were reported, including the prescription date, prescription duration, and prescription dose of the opioid. To extract the parameters of the HMM from the reported data, further observations—such as the cumulative count of opioid prescriptions for each patient, cumulative opioid doses, cumulative prescription duration, overlap for each prescription with the previous one, and the time interval between each prescription—also are calculated based on the reported data.

**Figure 2:**
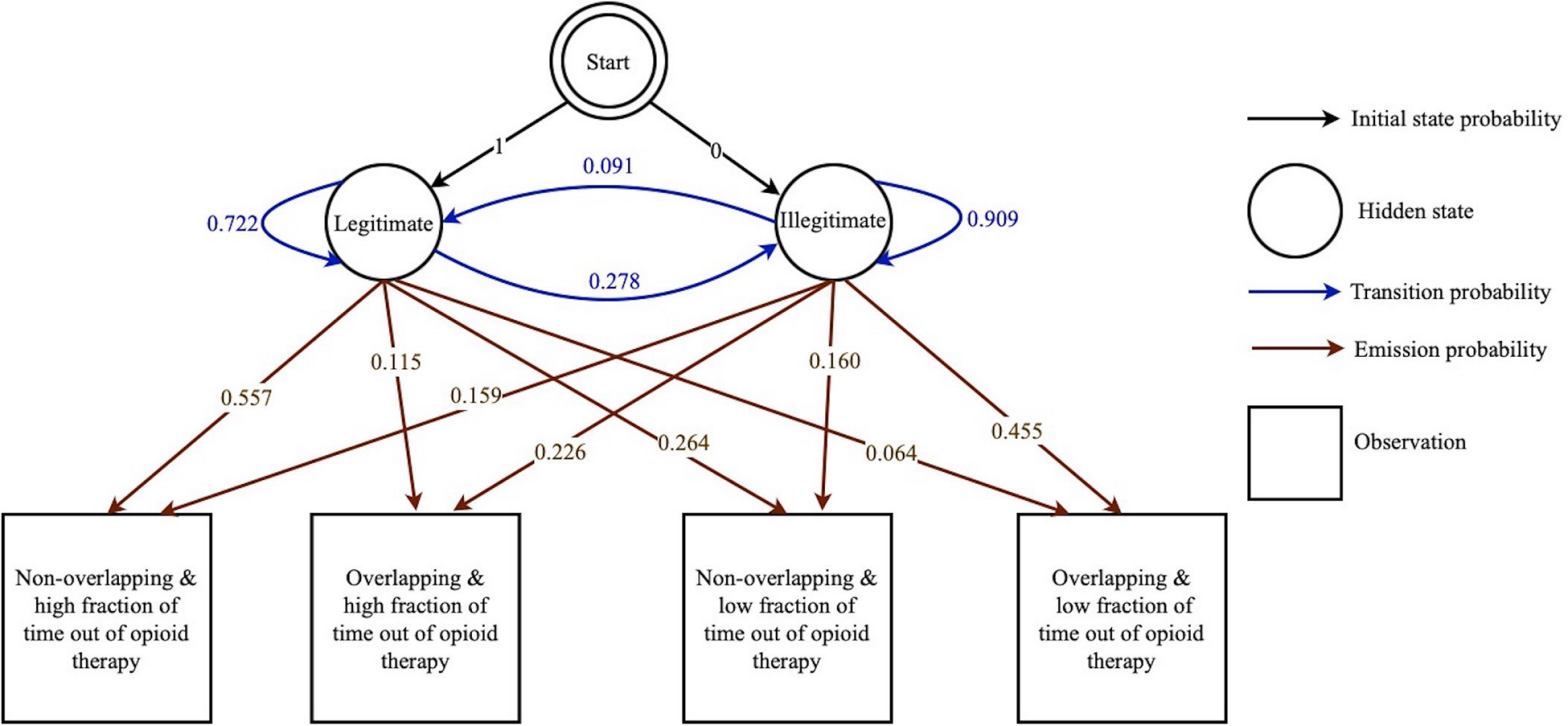
Graphical representation of the HMM-aided PDMP. This HMM-aided PDMP consists of initial state probabilities (represented by black arrows), hidden states (represented by circles), transition probabilities (represented by blue arrows), observations (represented by squares), and emission probabilities (represented by brown arrows).

An exploratory investigation of the longitudinal data of the patients finds that the “Legitimate” state and “Illegitimate” state of the prescription correspond to two distinct categorical distributions for dichotomous prescription overlap and fraction of time in which the patient was not on opioid therapy. This difference raises opportunities for machine learning-based classification of a given prescription refill attempt on the basis of these patterns. The opioid prescription overlap as the observation input was split into a vector of two sub-features as overlapping prescription and non-overlapping prescription. Furthermore, using the lower extreme of the box plot (disregarding outliers) of the fraction of time which legitimate patients were not on opioid therapy, the fraction of time which any patient was not on opioid therapy was also split into a vector of two sub-features less than and equal to the lower extreme and higher than the lower extreme. Finally, as a combination of these two separate categorical distributions, four observations for each state are defined. Using a package in R named “mHMMbayes” (70), the HMM parameters were estimated as depicted in Figure 2. The complete mathematical description of HMM algorithms and equations can be referenced in (71), while the discussion of the well-documented R package mHMMbayes can be found in (42, 72–74). In brief, the R package mHMMbayes fits the model by employing a hybrid Metropolis within Gibbs Markov Chain Monte Carlo algorithm. This approach extends the traditional HMM implementation by incorporating Bayesian estimation techniques (75). Therefore, the longitudinal data of various agents was studied using a multilevel HMM simultaneously, in which the aggregated level model was trained on agent level data with one overall categorical distribution (73).

Finally, the forward- backward algorithm was implemented in the agent-based model to compute the posterior marginals of two hidden state variables for any new prescription presented by a patient, given a sequence of previous observations of the prescription of the patient at the seeking prescription state. Then, based on the HMM-assumed thresholds, the prescription is classified as a legitimate or illegitimate opioid prescription. Illegitimate requests for opioid prescription cannot be filled; therefore, the person stops from entering the opioid therapy state in the opioid-prescribing state chart.

For each HMM threshold set between 0 to 1, with incremental step equal to 0.1 (in certain circumstances, in 0.01: see above), a Monte Carlo simulation for 10 years with 100 runs and a random seed was conducted. After the 5-year burn-in period for the model, the HMM-aided PDMP started to classify each prescription as a legitimate or illegitimate opioid prescription, and key metrics such as the cumulative number of correctly and incorrectly predicted positive cases, as well as the total positive cases and the total negative cases of illegitimate opioid prescription over 5 years, were reported. The initial analysis of these outcomes highlights that the HMM-aided PDMP is an imbalanced classification problem. Therefore, the HMM-aided PDMP performance with different HMM thresholds was evaluated *via* different metrics such as sensitivity (recall), specificity, concordance probability, accuracy, and F1 score. False negatives represent cases where the HMM-aided PDMP fails to identify illegitimate prescription refills, and mistakenly classifying them as legitimate. These instances pose a concern as they indicate missed opportunities to detect and intervene in potentially harmful situations. True positives refer to the correct identification of illegitimate prescription refills by the HMM-aided PDMP. These instances demonstrate the ability of the HMM-aided PDMP to accurately detect and flag suspicious behavior, aiding in preventing the misuse of prescription drugs. Minimizing false negatives and maximizing true positives are essential to ensure the effectiveness of the HMM-aided PDMP in accurately detecting illicit activities. Furthermore, false positives refer to cases where the HMM-aided PDMP incorrectly identifies legitimate prescription refills as illegitimate. These instances can lead to unnecessary interventions or delays for patients who legitimately require the prescribed medications. True negatives represent cases where the HMM-aided PDMP correctly identifies legitimate prescription refills. Minimizing false positives and maximizing true negatives are essential in ensuring that legitimate patients receive the medications they need without unnecessary interventions or delays. Sensitivity (i.e., recall) measures the ability of the HMM-aided PDMP to correctly identify true positives, that is, accurately detecting illegitimate prescription refills. A high sensitivity indicates that the HMM-aided PDMP has a strong capacity to capture instances of illicit activity, minimizing the risk of false negatives. Specificity evaluates the ability of the HMM-aided PDMP to correctly identify true negatives, referring to the accurate identification of legitimate prescription refills. A high specificity implies that the HMM-aided PDMP can effectively distinguish between legitimate and illegitimate cases, reducing the occurrence of false positives. Accuracy reflects how well the HMM-aided PDMP performs in correctly classifying both legitimate and illegitimate prescription refills. It considers the combined impact of true positives, true negatives, false positives, and false negatives. A higher accuracy indicates that the HMM-aided PDMP is making more correct predictions overall. Concordance probability measures the probability that the HMM-aided PDMP will rank a randomly chosen illegitimate refill higher than a randomly chosen legitimate refill. A high concordance probability indicates a strong discriminatory power of the HMM-aided PDMP, where it can effectively differentiate between the two classes. While the concordance probability focuses on the overall discriminatory power of the model, the F1 score considers the trade-off between precision and recall, and also providing insight into the overall performance of the HMM-aided PDMP. The F1 score is particularly valuable in scenarios involving imbalanced data sets, as is the case in this study where the emphasis lies on accurately rejecting illegitimate prescription fillings while ensuring legitimate prescriptions are filled. To provide additional information regarding precision, it can be stated that precision indicates the ability of the HMM-aided PDMP to accurately classify true positives while minimizing false positives. A high precision score implies that when the HMM-aided PDMP flags a refill as illegitimate, it is highly likely to be correct.

### 2.4. Interventions

To study the potential impact of reducing the opioid prescription doses and treatment durations, a simple PDMP and the HMM-aided PDMP on outcomes of interest, different interventions were examined. These interventions involved the following:

• Reducing the opioid prescription dose from baseline by 5%, 10%, 15%, 20%, and 25%.
• Reducing treatment duration from baseline by 5%, 10%, 15%, 20%, and 25%.
• Applying a simple PDMP that prevents filling overlapped opioid prescriptions.
• Applying the HMM-aided PDMP with four different HMM thresholds (i.e., 0.20, 0.30, 0.40, and 0.50) demonstrated a high F1 score, a high concordance probability, and a low false positive rate.
• Combinations of dual reductions in the prescription doses by 5%, 10%, 15%, 20%, and 25%, and treatment duration by 5%, 10%, 15%, 20%, and 25%.
• Combinations of any of the above interventions in which particularly strong benefits occurred when considered in isolation.

For each intervention or combined interventions, a Monte Carlo simulation with 100 realizations was conducted to ensure that a broad set of values for the parameters treated as random variables was drawn from distributions. To accommodate transients associated with the initial state, each simulation employed a 5-year burn-in period for the model. Following the burn-in period, the model was run for a time horizon of 5 years more to track outcomes of interest for each run. Finally, the result section reported percentage change of medical and non-medical pharmaceutical opioid use-related overdoses, percentage change of street opioid initiation, and percentage change of legitimate, illegitimate, and total filled opioid prescription from the baseline over 5 years for each intervention or selected combined interventions. These outcomes were compared across interventions to identify the most effective intervention for reducing the harms associated with prescription opioid use.

## 3. Results

The baseline scenario yields approximately 326,500 opioid prescription fills with 3% illegitimate requests for prescription over 5 years. Meanwhile, 18% of the patients prescribed with opioids in the model misuse them at any given time, and 4% of the patients who misuse prescription opioids eventually transition to street opioids. The total number of opioid overdoses is approximately 200 over 5 years, which implies that the prevalence of overdose among patients treated with prescribed opioids is 0.03%.

### 3.1. Single interventions

#### 3.1.1. Lowering the prescription doses

Supplementary Table S6 shows the results of lowering the prescription doses for the 5-year outcomes of interest. Over 5 years, lowering the prescription doses by 5% would provide a 0.25% reduction in medical overdose; with a 15% and 25% reduction in dose, the effect changes to 5.32% reduction and 11.41% reduction in medical overdoses, respectively.

Lowering the prescription doses has also a favorable effect on non-medical overdoses. Over 5 years, lowering the prescription doses by 5% would provide a 1.27% reduction in non-medical overdose: with a 15% and 25% reduction, the impact changes to 5.02% reduction and 12.17% reduction in such overdoses.

Lowering the prescription doses slightly decreases the total number of individuals who escalate to heroin over 5 years (by a 1.65% decrease and 3.85% decrease in such escalation for a 15% and 25% dose reduction, respectively). Moreover, lowering the prescription doses would have a modest effect on reducing the total number of filled opioid prescriptions, yielding a reduction by 0.05% and 0.30% for a 15% and 25% lowered dose, respectively, over 5 years. It further secures a reduction in illegitimate opioid prescriptions (reducing such prescriptions by 2.61% and 6.86% for a 15% and 25% lower dose, respectively, over 5 years). It is notable that such results exhibit an elasticity of effect, leading, for example, to a doubling of the reduction in dose more than doubling the benefits in certain outcomes of interest. Such effects suggest the value of a closer examination of the dependence of such results on dose changes.

#### 3.1.2. Lowering treatment duration

Supplementary Table S6 shows results of reductions in treatment duration on the 5-year outcomes of interest. Lower treatment durations have a larger favorable impact on both medical and non-medical overdoses over 5 years. Lowering treatment duration in the model by 15% yields a reduction in medical overdose and non-medical overdose of 7.59% and 14.33%, respectively, over 5 years. Lowering treatment duration by 25% would provide a 14.47% reduction in medical overdose and 27.13% reduction in non-medical overdose, respectively, over 5 years.

Such benefits must be counterbalanced with the fact that lowered treatment duration in the model imposes the worst outcome in terms of the total number of individuals who escalate to heroin over 5 years. Specifically, 15% and 25% lower treatment duration in the model yields a 6.01% increase and 8.24% increase escalation to heroin over that half-decade, respectively.

A lowered treatment duration also results in an increase in the number of filled opioid prescriptions over 5years, with a 3.83% increase and 6.75% increase in such filled prescriptions resulting from a 15% and 25% lowering in treatment duration, respectively, over 5 years. Reduced treatment duration also leads to a superlinear increase in illegitimate opioid prescriptions by 4.85% and 10.52% for a 15% and 25% lowering in treatment duration, respectively, over 5 years.

#### 3.1.3. Prescription drug monitoring program

In order to examine the effectiveness of the HMM-aided PDMP, the ROC curve and associated area under the curve (AUC) for different HMM thresholds (see Supplementary Figure S8) and the precision-recall curve and associated AUC for different HMM thresholds (see Supplementary Figure S9) were calculated. Plausibly acceptable frequencies of positive and negative results of the HMM-aided PDMP across both ROC and the precision-recall curves can be achieved with HMM thresholds equal to 0.2, 0.3, 0.4, and 0.5. This set of HMM thresholds is located progressively closer to the upper left-hand corner in the ROC curve plot and the upper right-hand corner in the precision-recall curve plot that reflects the progressively greater discriminant capacity of the HMM-aided PDMP. In addition, they exhibit a high F1 score, a high concordance probability, and a low false positive rate compared with other HMM thresholds (see Supplementary Table S5). The corresponding AUCs confirm that the HMM-aided PDMP has a suitable predictive ability to differentiate illegitimate prescription refills from legitimate prescription refills.

Supplementary Table S7 shows results for a simple PDMP (i.e., blocking the acquisition of overlapping prescriptions) and the HMM-aided PDMP interventions. The simple PDMP has only a small effect on medical and non-medical overdose (yielding a 0.24% increase and 0.59% reduction, respectively). By contrast, the simple PDMP exerts a large reduction in illegitimate opioid prescriptions (41.89% over 5 years), leading to a massive number of individuals who cannot access opioid prescriptions and who therefore escalate to street opioids (resulting in a 101.97% increase over 5 years). While the HMM-aided PDMP shares only a small impact on medical overdose (by 0.63% increase and 0.24% increase for 0.2 and 0.4 HMM- threshold, respectively), the HMM-aided PDMP has a larger impact on non-medical overdose (yielding a 2.11% reduction and 4.20% reduction for HMM-thresholds of 0.2 and 0.4, respectively). Compared with the Baseline, the HMM-aided PDMP also achieves notable decreases in illegitimate opioid prescriptions, with 0.2 and 0.4 HMM thresholds yielding reductions of 44.30% and 43.45%, respectively, over 5 years. However, the advantages of the HMM-aided PDMP are subject to a crucial side effect shared with its simple PDMP counterpart: significant increases in the number of individuals who shift to street opioids. Specifically, the HMM-aided PDMP precipitates 105.43% and 94.97% increases in transitions to street options for HMM thresholds of 0.2 and 0.4, respectively.

### 3.2. Combined interventions

Supplementary Table S8 shows results for combinations of two interventions—lowered prescription doses and lowered treatment duration—with different levels of reduction. The most favorable impact in reducing three outcomes of interest (cumulative count of medical and non-medical overdoses and count of individuals who escalate to street opioids) was achieved by lowering treatment duration by 5% combined with lowering the prescription dose by either 20% or 25%. Illegitimate opioid prescriptions also decreased with these combinations.

Strong reductions in medical and non-medical overdose would also be achieved with the combination of a 25% reduction in the prescription dose with lowering treatment duration either by 10% or 15%; however, this combination also leads to a slight increase in individuals who escalate to street opioids.

Supplementary Table S9 shows results for three interventions, each representing a combination of interventions in which particularly strong benefits occurred when considered in isolation. These combined interventions are the following: (1) the combination of lowering the prescription doses by 25% and the HMM-aided PDMP with threshold equal to 0.40; (2) the combinations of lowering treatment duration by 25% and the HMM-aided PDMP threshold equal to 0.40; and (3) the combination of lowering treatment duration by 10%, lowering the prescription doses by 20%, and the HMM-aided PDMP threshold equal to 0.40. In all three cases, intervention combinations are less beneficial than the lowering the prescription doses or treatment durations by corresponding amounts in isolation and leading to nearly two times higher escalation to street opioids relative to the baseline.

## 4. Discussion

This work sought to secure both methodological and public health insights from the use of a machine learning-equipped stylized agent-based simulation model characterizing dynamics associated with prescription opioid use and the risk of shifts to street-sourced opioids. While this work evaluated the accuracy extending from using the hidden Markov model to recognize the individuals engaged in opioid seeking for legitimate needs, it did so in the broader context of a simulation model recognizing the risk that the individuals flagged as engaged in doctor shopping would transition to street opioid use, and of overdoses. As an example of the application of this framework, this work investigated the opioid overdose and street opioid escalation impacts of two policies targeting opioid prescription practices among patients prescribed with pharmaceutical opioids.

The majority of the data utilized in this work was sourced and derived from a study published by the Canadian Institute for Health Information (48), a Canadian Federal institution that seeks to preserve public trust by placing foremost importance on ethical considerations and responsible data handling practices. In its commitment to the Canadian public, CIHI is committed to protecting patient privacy, ensuring data security, addressing biases in data collection and analysis, and promoting transparency and accountability (76). Furthermore, we carefully documented and detailed the assumptions made during the development of the simulation model to ensure transparency and provide a clear understanding of the underlying principles. We additionally employed rigorous validation procedures to assess the performance of the simulation model and assess potential biases.

At a methodological level, the findings suggest both the practicality and desirability of informing agent-based models using machine learning methods at an individual level. This work further demonstrated that the simple and computationally frugal approach of the hidden Markov modeling can achieve favorable accuracy profiles, raising the potential for more heavily data-driven approaches to further boost both the accuracy of the classification and the public health gains from HMM-informed policies.

With respect to the example policies examined here, the simulation model findings suggest that if used aggressively to lower doses, lowering the prescription doses could have the most favorable impact on the outcomes of interest over 5 years while minimizing burden on the patients with a legitimate need for pharmaceutical opioids.

Lowering treatment duration would introduce varying degrees of potential unintended consequences by escalating some patients who cannot access prescribed pharmaceutical opioids to street opioids. These unintended consequences vary widely across different types of intervention, especially with the PDMP. Both the simple PDMP and the HMM-aided PDMP readily reduced the number of illegitimate opioid prescriptions and decreased the supply of prescription opioids, but thereby caused some patients to shift to street opioid use.

Note that a combination of lowering in prescription doses and lowering in treatment duration did not perform much better than lowering the prescription doses, considered alone, with the respect to all outcomes of interest. Moreover, the combination of the HMM-aided PDMP with other interventions presented above are only examples of the more than 100 possible combinations of strategies, and in the exploratory testing of other combinations of strategies, the adverse influence of the HMM-aided PDMP on escalation to the street opioids is always a pronounced dynamic.

The current study offers two primary findings. The first is that it is misleading to consider combined interventions to always provide a greater positive effect on the potential issue than any single policy. While the combined interventions exert a large positive impact on reducing both medical and non-medical opioid overdose, when considered in terms of reducing the number of individuals who shift to street opioids, none of the combined interventions achieved more favorable outcomes than the single intervention of lowering in opioid dose by 25%. Ultimately, it is essential to create a comprehensive set of outcomes to test the multi-dimensional effect of the suggested interventions.

The second major finding is the strong benefits conferred by jointly conducting machine learning and agent-based modeling when using the latter to understand the downstream consequences and characterize the dynamic context for the former. Within the application examined here, the individual-level simulation model incorporated machine learning as a component of individual-level policy targeting, with the agent-based model characterizing both the patient–provider encounters that required such evaluation, and the subsequent evolution of the patient trajectory, including their experience of adverse events. While other studies have examined the prescription or short-term outcome classification accuracy achieved by machine learning strategies on the records of prescription drug use (77–80), the current modeling analysis is the first to demonstrate that the HMM-aided PDMP could be more beneficial in reducing overdose among patients who have been prescribed with opioids than simple PDMP. More significantly, beyond the use of a simulation model serving to produce a set of well-grounded data to test the possibility of applying machine learning to healthcare problems, this implementation demonstrates that a simulation model can evaluate the trade-offs involved in the application of machine learning algorithms in healthcare more broadly and in more textured contexts than is traditionally pursued, by evaluating the impact of healthcare machine learning-supported decisions within and on the life course of the patient.

This study is encumbered by a number of notable limitations. The scope of the model excludes consideration of measures that would reduce the necessity of continued opioid use, such as through the use of alternative pain management techniques. Furthermore, the current model differentiates neither between fatal and non-fatal overdoses, nor between accidental and intentional overdoses. However, based on adherence of the patient to opioid treatment, overdoses are classified into two categories: medical and non-medical overdoses. This classification offers strong benefits in evaluating the suggested interventions. Moreover, the current model does not consider the uses of illegitimate opioid prescriptions to obtain pharmaceutical opioids for resale; within the current work, such prescriptions are considered as being solely intended for personal use. The hidden Markov model seeking to address the diversity of opioid use behaviors in a broader empirical context may require additional latent states and transitions to capture the distinct patterns of opioid seeking and transition dynamics that are observed in this broader context. The scoping of this part of the agent-based model may serve to partly compensate for the model's exclusion of consideration of other avenues of accessing diverted opioids, such as pharmaceutical opioids that were lost or stolen from community pharmacies, companies, or hospitals.

The current simulation model can render less stylized and by providing it with stronger empirical grounding data about pharmaceutical opioid use from other sources, including untraditional forms of data, such as from social media and wastewater surveillance systems. Moreover, extending the model domain to capture street drug use, criminal justice involvement, and social influence on illegitimate opioid prescription seeking and misuse of diverted pharmaceutical opioids could help support more granular estimates of the effects of the suggested interventions examined here. Finally, a model representation of other components of the heterogeneity of the patients in the simulation model including sex and other demographics, history of trauma, mood and anxiety disorders, and the duration of pain complaints and pain location may have a positive impact on the discriminant capacity of an HMM-aided PDMP.

This study offers some support for possible policy avenues to lessen the distressingly heavy burden the opioid crisis has imposed on the Canadian population. However, there is a large population of individuals with existing opioid use disorders, and while the size of that population notably limits securing the full potential benefit of the policies studied here, it also emphasizes the need to ensure efficient prioritization and use of the limited flow of resources available for preventive strategies. Thus, while stylized, the current findings may offer steps toward aiding the public health community in enhancing the effectiveness of measures focused on preventing the development of opioid use disorders *via* prescription drug pathways.

## Data Availability

The original contributions presented in the study are included in the article/**Supplementary Material**, further inquiries can be directed to the corresponding author.

## Footnotes

^1^“Legitimate medical purpose means a therapeutic treatment regimen or program generally recognized and accepted in the field of medical science as being safe and effective in the diagnosis, treatment, correction, or alleviation of the specific medical condition of the patient under all relevant circumstances” (62).
