# Supplementary material for: Opioid-related harms and care impacts of conventional and AI-based prescription management strategies: insights from leveraging agent-based modeling and machine learning
Source: Front Digit Health. 2023 Jun 20;5:1174845. doi: 10.3389/fdgth.2023.1174845 (PMC10318360; doi:10.3389/fdgth.2023.1174845)
Supplement: Supplementary file 1 [file Datasheet1.docx]

Supplementary Material

**Contents**

Figure S1. Opioid Prescribing state chart.

Figure S2. Medication Adherence state chart.

Table S1. List of parameters for opioid therapy agent-based model.

Table S2. List of assumptions for opioid therapy agent-based model.

Figure S3. The distribution and moving average of the number of people in an individual's social circle.

Table S3. List of parameters used in opioid therapy agent-based model calibration and validation.

Table S4. List of parameters that are calibrated and the corresponding empirical data used for the calibration process.

Figure S4. (a) The proportion of people starting opioids without being prescribed opioids in the past year, and (b) the proportion of people prescribed opioids.

Figure S5. (a) The proportion of patients prescribed opioids who either underuse or overuse them, and (b) the proportion of patients who overuse them

Figure S6. (a) The proportion of people prescribed opioids who were on long-term therapy, and (b) the proportion of patients who misuse prescription opioids transition to street opioid.

Figure S7. The prevalence of illegitimate prescriptions.

Figure S8. ROC curve plot and associated AUC for different HMM thresholds.

Figure S9. Precision-recall curve plot and associated AUC for different HMM thresholds.

Table S5. List of machine learning metrics for HMM-aided PDMP with different HMM thresholds.

Table S6. Results for single interventions.

Table S7. Results for the simple PDMP intervention and HMM-aided PDMP interventions.

Table S8. Results of combinations of dual reductions in prescription doses and treatment duration.

Table S9. Results of combinations of HMM-aided PDMP, lowering in prescription doses and lowering in treatment duration.

#
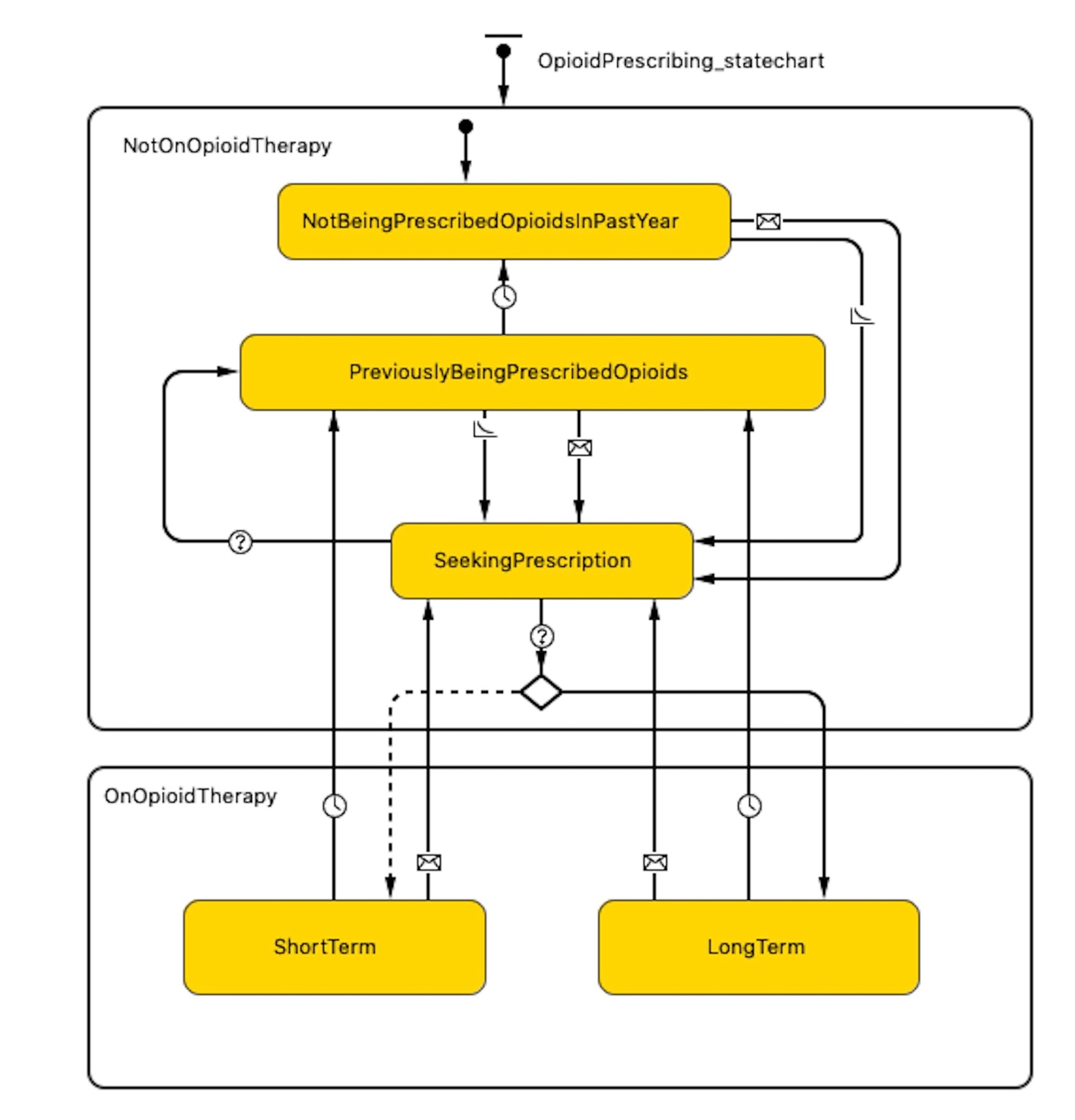


Figure S1. Opioid Prescribing state chart. This state chart seeks to depict states observable from the perspective of health care providers.


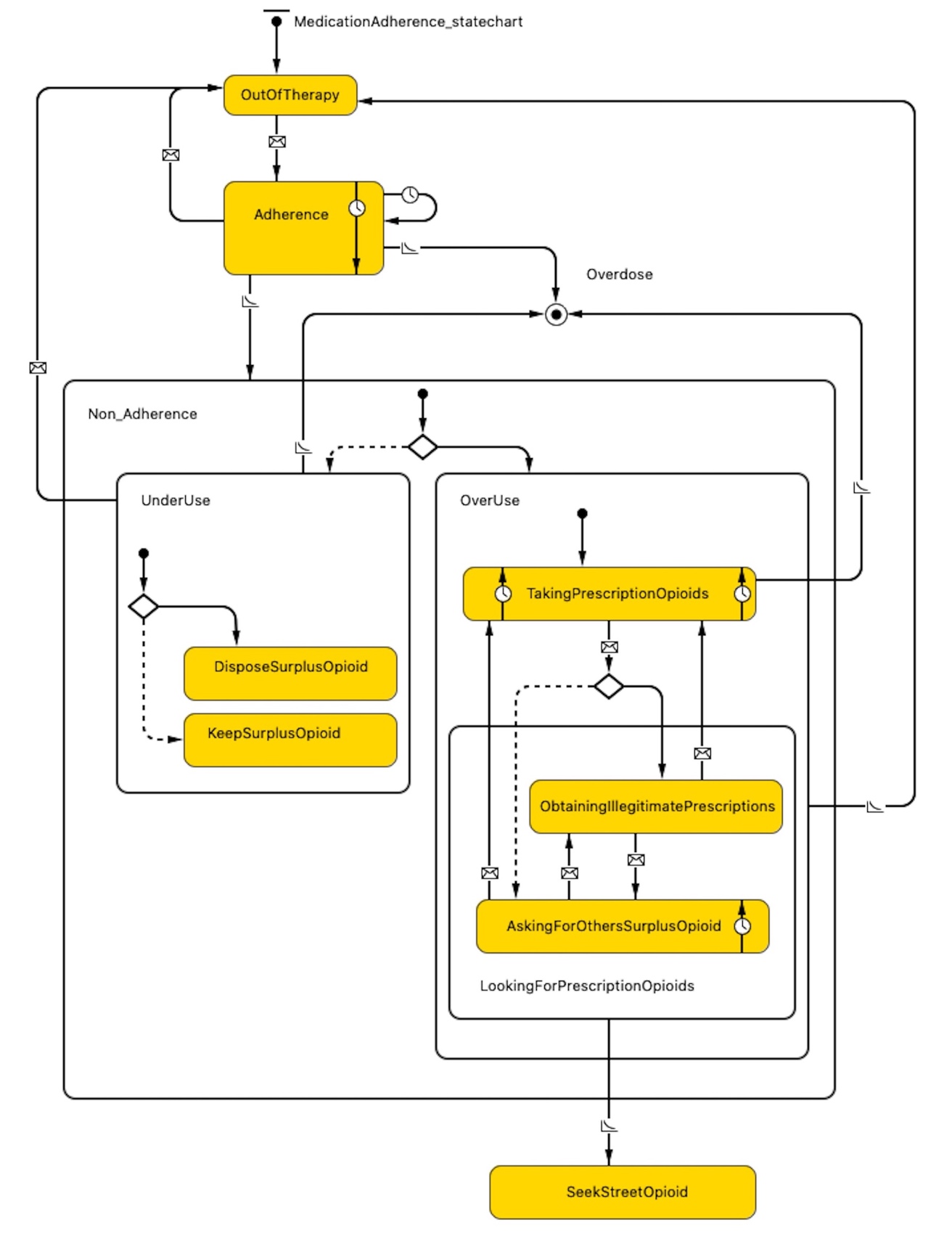


Figure S2. Medication Adherence state chart. This state chart seeks to depict states that are unobservable from the perspective of health care providers.

Table S1. List of parameters for opioid therapy agent-based model

| Parameter | Description | Values | References |
| --- | --- | --- | --- |
| Population size (Persons) | Population size at the model’s initialization. | 50000 | Assumed^1^ |
| Duration of use for people starting opioids | Represents prescription opioid use duration for people without opioid therapy in the past year | Custom Distribution (Maximum time is calibrated to give rise to the proportion of people prescribed opioids who were on long-term therapy) | Parametrized CIHI (1) |
| Doses being prescribed to people starting opioids (by average MMEs^3^ daily) | Represents prescription opioid use doses for people without opioid therapy in the past year | Custom Distribution | Parametrized CIHI (1) |
| Duration of use for established patients | Represents prescription opioid use duration for people with opioid therapy in the past year | Custom Distribution (Maximum time is calibrated to show proportion of people prescribed opioids who were on long-term therapy) | Assumed^2^ based on CIHI (1) |
| Doses being prescribed to patients on long term therapy (by average MMEs daily) | Represents prescription opioid use doses for patients with current prescription opioid use duration more than 90 days | Custom Distribution | Parametrized CIHI (1) |
| Doses being prescribed to established patients on short term therapy (by average MMEs daily) | Represents prescription opioid use doses for people with opioid therapy in the past year and current prescription opioid use duration less than 90 days | Custom Distribution | Assumed^2^ based on CIHI (1) |
| Opioid prescription rate for new patients (1/ Week) | Represents rate per week of new patients prescribed opioid. | 0.0005 | Calibrated to CIHI (1) |
| Opioid prescription rate for established patients (1/ Week) | Represents rate per week of established patients prescribed opioid. | 0.06 | Calibrated to CIHI (1) |
| Initial amount of nonadherence rate (1/Week) | Represents initial rate per week of loss of adherence to opioid treatment. | 0.0335 | Parametrized (2) |
| Loss of adherence coefficient | Represents growth rate per week for nonadherence rate | 0.035 | Calibrated to (3) |
| Probability overuse opioid after the loss of adherence | Probability that a patient starts overusing prescription opioids after the loss of adherence | 0.235 | Parametrized (4) |
| Opioid dose accretion coefficient | Represents growth rate per week of required opioid doses for patients in the overuse state | 0.0008 | Calibrated to (3) |
| Self-care-treatment rate (1/Week) | Represents rate per week of stopping overusing opioid due to self-care or treatment | 0.06 | Calibrated to (3) |
| Probability attempting to obtain illegitimate prescriptions when in overuse state | Probability that a patient in an overuse state attempt to obtain illegitimate prescriptions | 0.5 | Assumed^2^ |
| Transition rate to street opioid for patients who misuse prescription opioids with no access to prescription opioids (1/ Week) | Represents rate per week of transition to street opioid for patients who misuse prescription and cannot obtain prescription opioids | 0.4 | Calibrated to (3) |
| Probability of dispose of all surplus opioid | Probability that a patient in the underuse state disposes of all surplus opioid | Between 0.05 and 0.25 | Parametrized (5) |
| Available surplus of prescription opioids | The portion of prescription opioids which have been stored by each patient in underuse state | Between 1 and 1/ 3 | Assumed^2^ |
| Overdose rate based on recent opioid doses (1/Year) | Represents rate per year of overdose occurrence for patients currently using opioids | Custom Distribution | Parametrized (6) |

1-The coefficient of variation across different population sizes remains less than 10% for all outcomes, except for nonmedical overdoses, which remain less than 15%. This observation suggests that variations in the current population size have minimal impact on the overall outcome.

2- The assumed parameters are derived from the researcher's judgment, literature review, domain expertise, and assumptions made about the underlying system.

3-MMEs: Morphine milligram equivalents

Table S2. List of assumptions for opioid therapy agent-based model

| Assumption and Simplification | Justification |
| --- | --- |
| The simulation model follows a population cohort. | Model run time is short (5 years burn-in and 5 years results analysis) |
| Reference data is for three Canadian provinces cumulatively (Ontario, Saskatchewan, and British Columbia). | Currently available data for the individual cities are limited, therefore the patterns of results are studied for these three provinces cumulatively as well. |
| As time passes, patients in opioid therapy are more likely to lapse in adherence to opioid treatment | Based on the related literature (7,8), the loss of adherence coefficient is calibrated to the reference data (see Table S1) |
| After entering an overuse state, the need for the opioid dose increases exponentially over time. | Based on the related literature (9,10), the opioid dose accretion coefficient is calibrated to the reference data (see Table S1) |
| Misusing opioid behavior is divided into two mutually exclusive situations: overuse or underuse. | If overuse behavior is dominant, patients will not have any surplus opioid for sharing |
| Illegitimate prescription requests are identified by pharmacists at pharmacies. | The Prescription Monitoring Program (PMP) was initially implemented at pharmacies and used by Pharmacists (11). |
| If patients in an overuse state try to obtain surplus opioids from others, they will get all available surplus opioids. | Outcomes of the simulation model are not sensitive to this simplifying assumption. |
| Patients in the underuse state store different portion of their surplus opioids. | Outcomes of the simulation model are not sensitive to this simplifying assumption. |

**
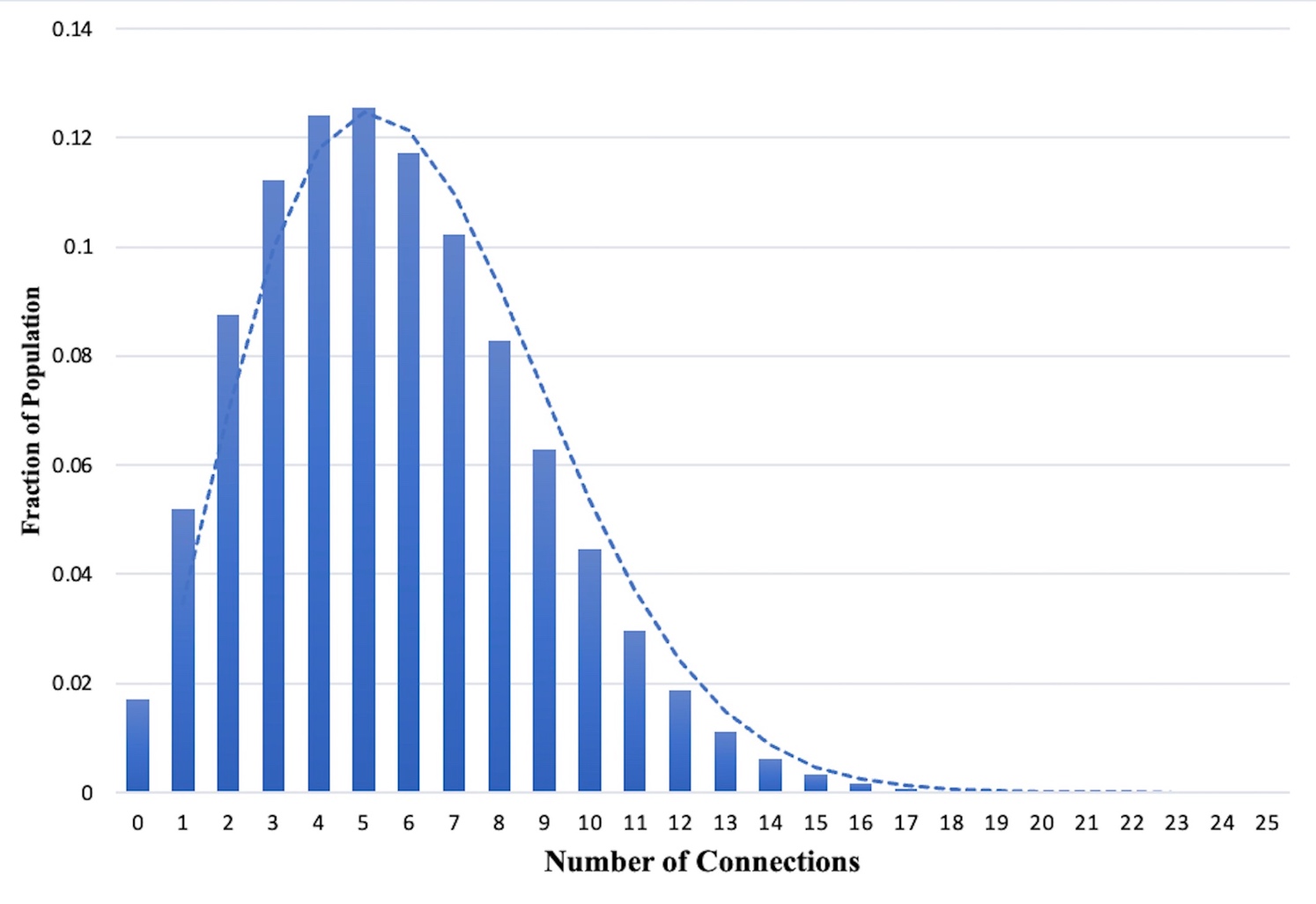
**

Figure S3. The distribution and moving average indicated by bar chart and dashed curve, respectively, of the number of people in an individual's social circle. The x-axis represents the number of people an individual may know, and the y-axis shows the fraction of the population with a given number of people in their circle.

Table S3. List of parameters used in opioid therapy agent-based model calibration and validation

| Parameter | Values | References |
| --- | --- | --- |
| Proportion (%) of the study population starting opioids without being prescribed opioids in the past year | From 9.5% in 2013 to 8.1% in 2018 | Based on CIHI (1) |
| Proportion (%) of people prescribed opioids | From 14.26 % in 2013 to 12.32% in 2018 | Calculated^1^ based on CIHI (1) |
| Proportion (%) of people prescribed opioids who were on long-term therapy | From 19.8% in 2013 to 17.6% in 2018 | Based on CIHI (1) |
| Prevalence of successful opioid doctor shopping as the behavior of visiting different prescribers and/or pharmacies to obtain opioids | Between 0.2% to 4% | Based on (12) |
| Proportion (%) of patients prescribed opioids misuse them | Between 21% to 29% | Based on (3) |
| Proportion (%) of patients who use opioids and develop opioid use disorder | Between 8% to 12% | Based on (3) |
| Proportion (%) of patients who misuse prescription opioids transition to heroin | Between 4% to 6% | Based on (3) |

1-The total percentage of the interested parameter for three Canadian provinces was computed by multiplying the individual percentages of the parameter in each province by their respective total population. After summing these values, the total parameter value was divided by the total population across all three provinces and multiplied by 100 to obtain the percentage.

**Table S4. List of the parameters that are calibrated and the corresponding empirical data used for the calibration process.**

| Parameters which are calibrated | Data against which calibration occurs (see Table S3 for references) |
| --- | --- |
| Opioid prescription rate for new patients (1/Week) | Proportion (%) of the study population starting opioids and Proportion (%) of people prescribed opioids |
| Opioid prescription rate for established patients (1/ Week) | Proportion (%) of the study population starting opioids and Proportion (%) of people prescribed opioids |
| Loss of adherence coefficient | Proportion (%) of patients prescribed opioids misuse them |
| Opioid dose accretion coefficient | Proportion (%) of patients who use opioids and develop opioid use disorder |
| Self-care-treatment rate (1/Week) | Proportion (%) of patients using an opioid develop an excessive opioid use |
| Maximum time of duration of use for people starting opioid | Proportion (%) of people prescribed opioids who were on long-term therapy and Proportion (%) of the study population starting opioids and Proportion (%) of people prescribed opioids |
| Maximum time of duration of use for established patients | Proportion (%) of people prescribed opioids who were on long-term therapy and Proportion (%) of the study population starting opioids and Proportion (%) of people prescribed opioids |
| Transition rate to street opioid for patients who misuse prescription opioids with no access to prescription opioids | Proportion (%) of patients who misuse prescription opioids transition to heroin and Prevalence of successful opioid doctor shopping |


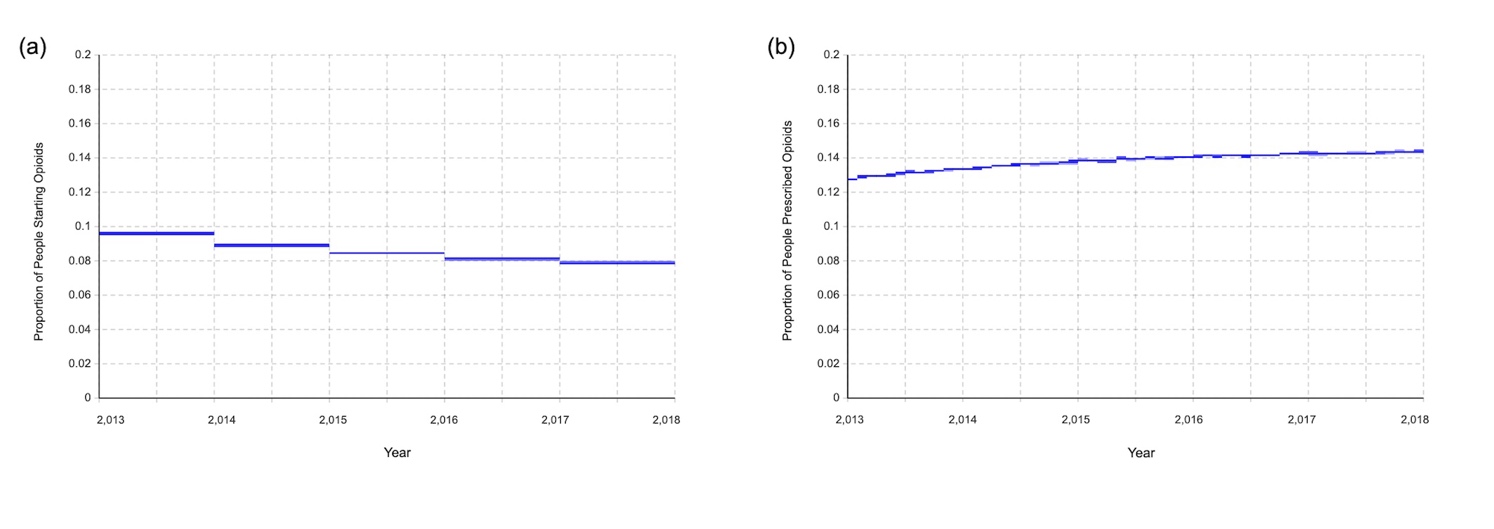
Figure S4. (a) The proportion of people starting opioids without being prescribed opioids in the past year and (b) the proportion of people prescribed opioids, for the baseline scenario generated by the model fitted well with the references data (see Table S3 for the references data).


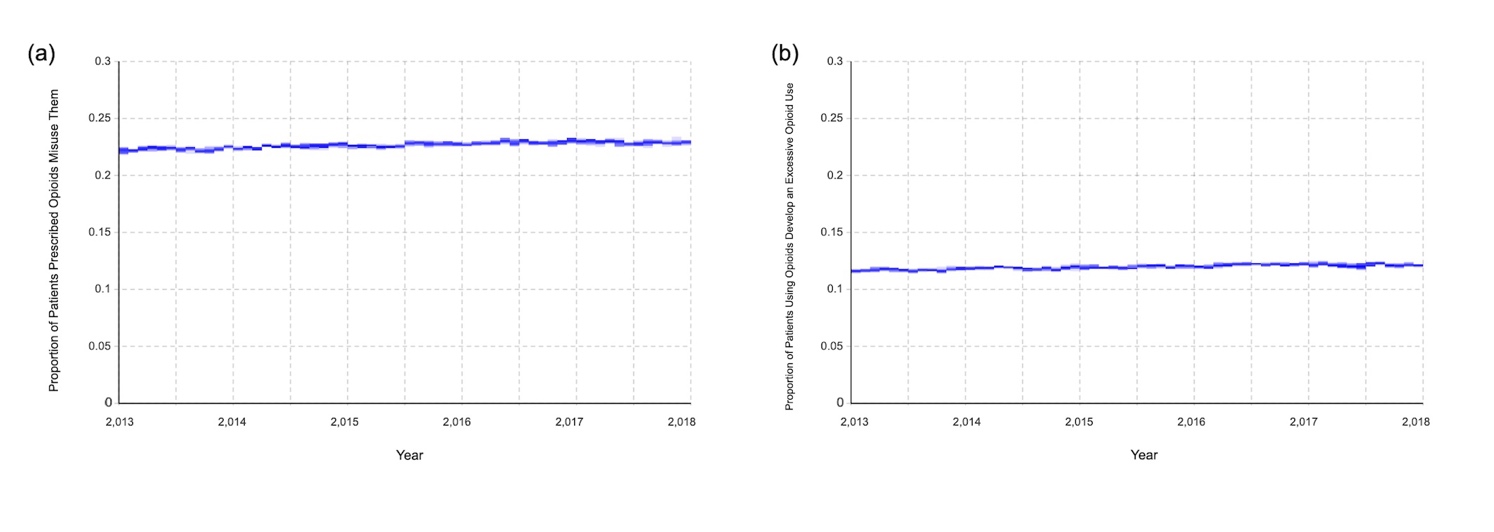


Figure S5. (a) The proportion of patients prescribed opioids who either underuse or overuse them, and (b) the proportion of patients who overuse them, for the baseline scenario, generated by the model fitted well with the references data (see Table S3 for the references data).


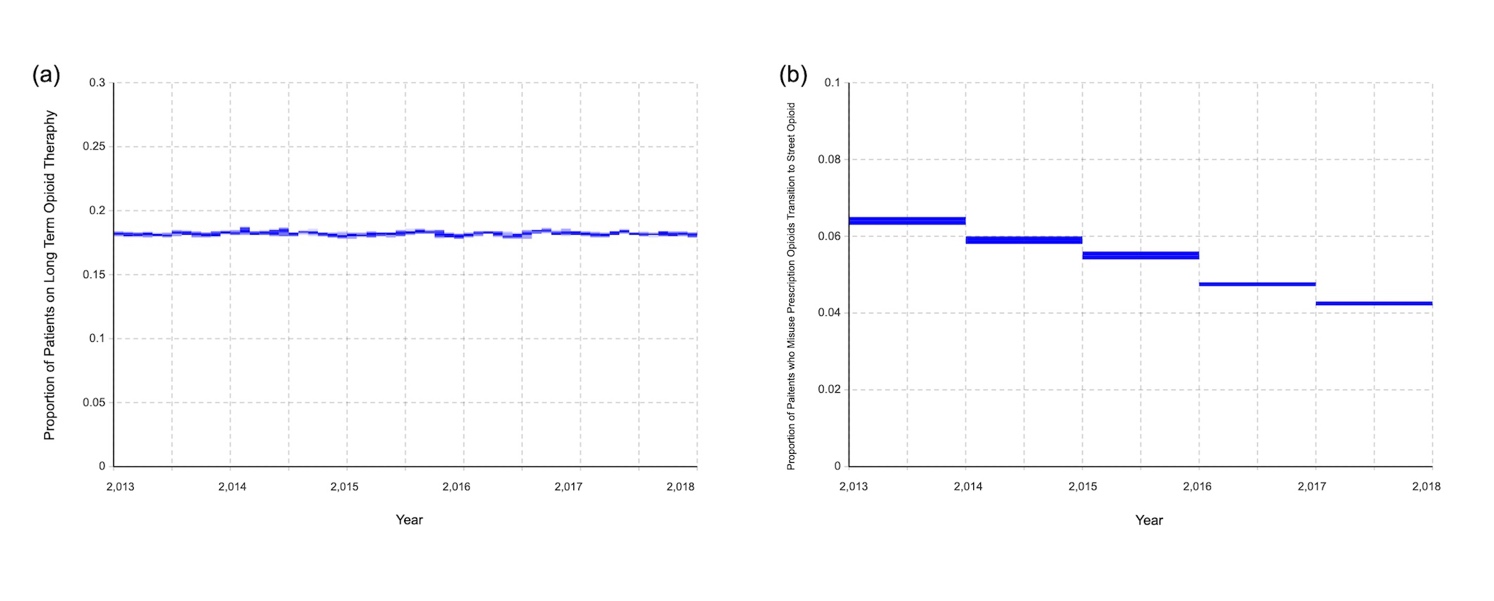


Figure S6. (a) The proportion of people prescribed opioids who were on long-term therapy, and (b) the proportion of patients who misuse prescription opioids transition to street opioid, generated by the model fitted well with the empirical data (see Table S3 for the references data).


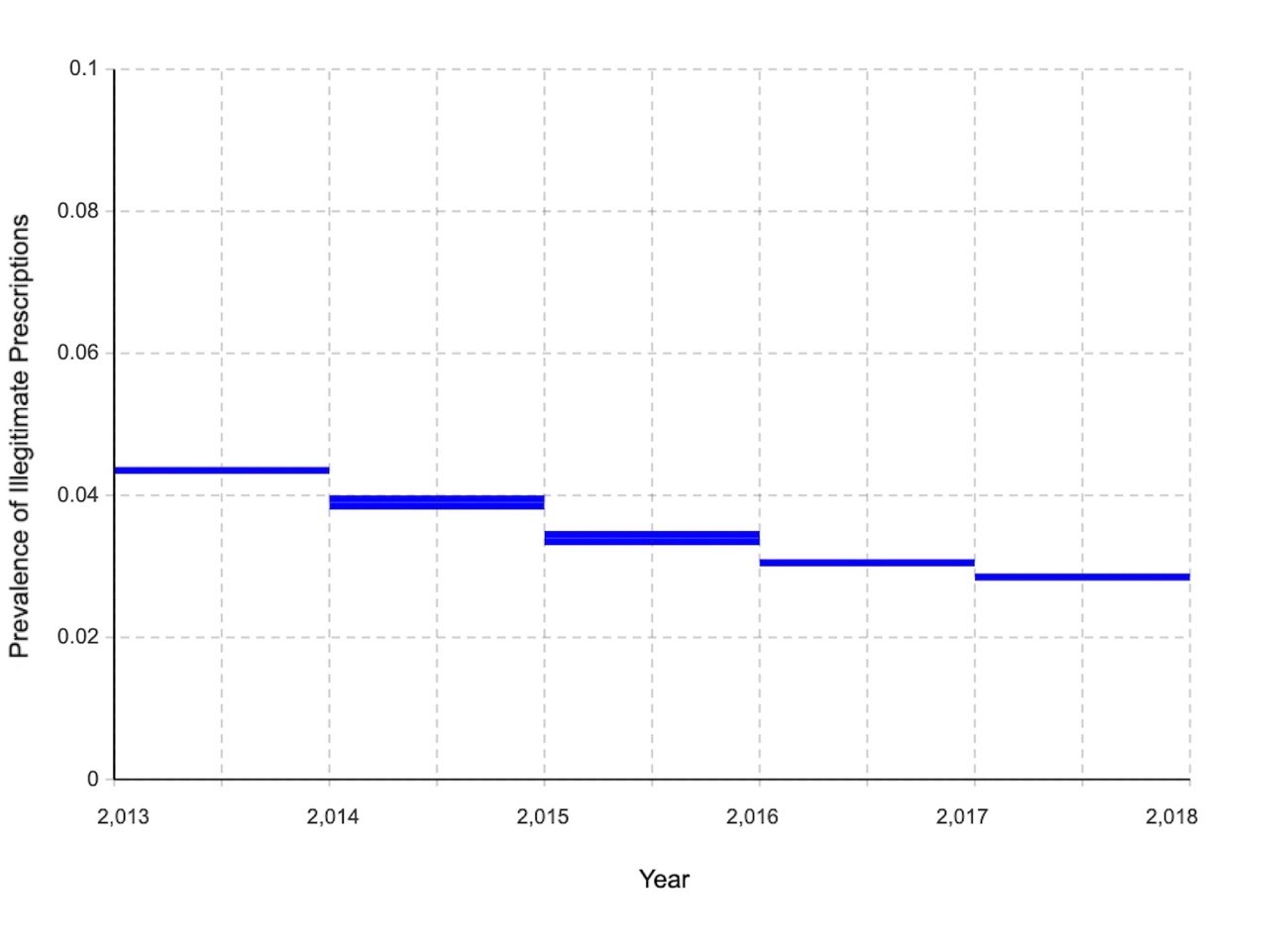


Figure S7. The prevalence of illegitimate prescriptions (e.g., doctor shopping), generated by the model fitted well with the empirical data (see Table S3 for the references data).


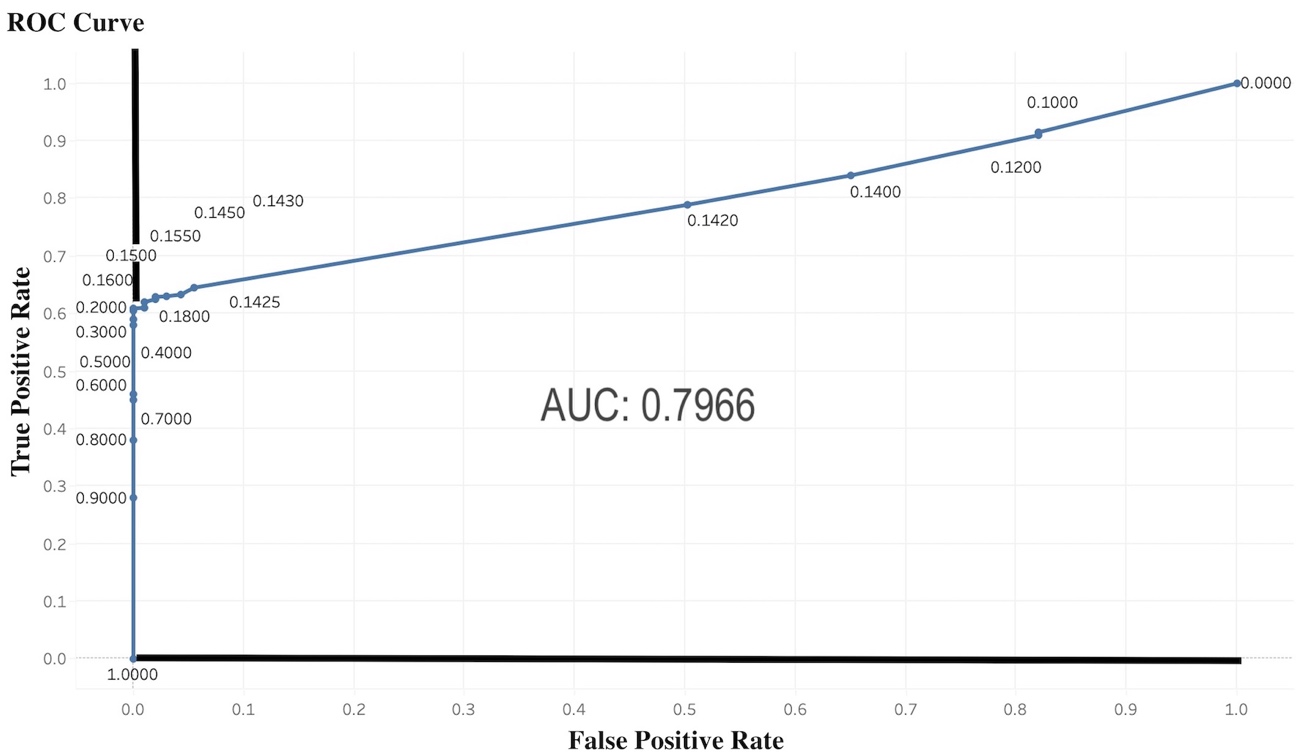


Figure S8. ROC curve plot and associated AUC for different HMM thresholds (shown as number labels along-side the curve) within the HMM-aided PDMP. A no-skill classifier for PDMP which is no better than chance lies on the diagonal line on the ROC curve plot.


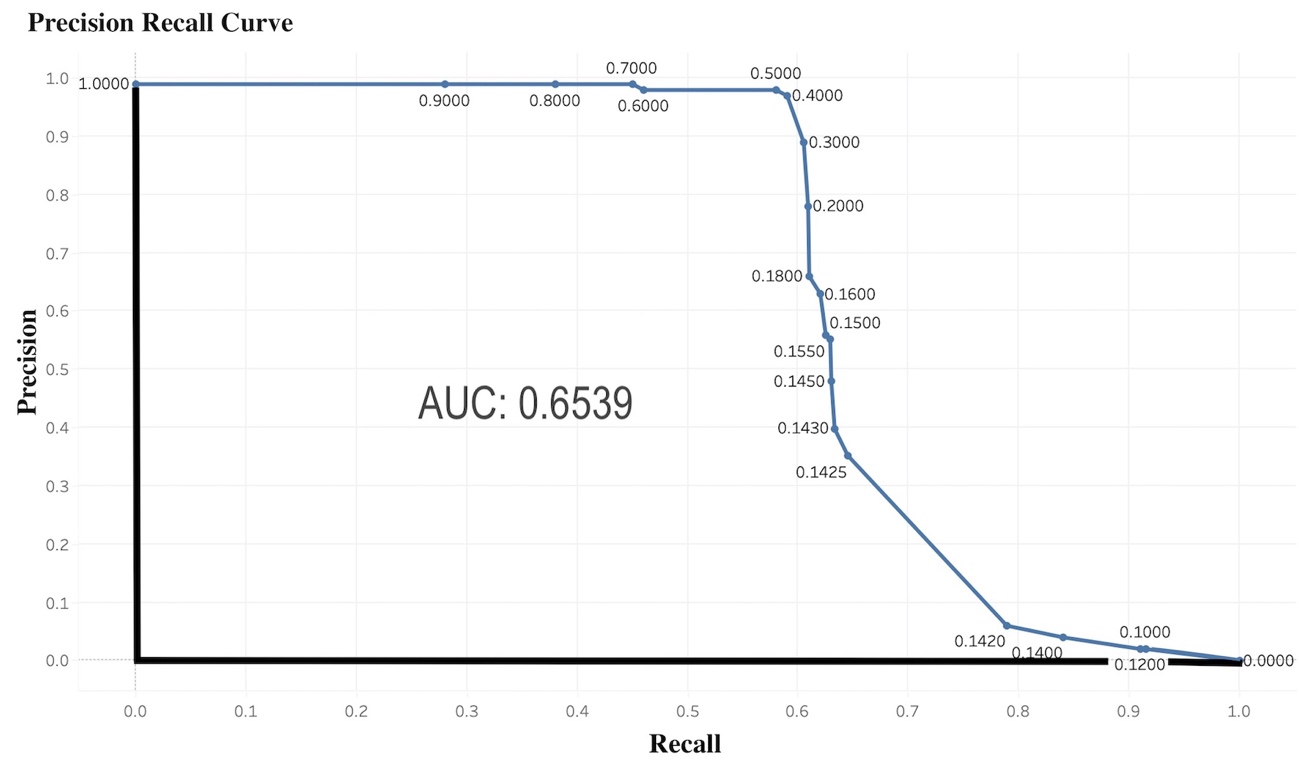


Figure S9. Precision-recall curve plot and associated AUC for different HMM thresholds (shown as number labels along-side the curve) within the HMM-aided PDMP. A no-skill classifier for PDMP which is no better than chance lies on a horizontal line on the precision-recall curve plot with a precision equal to ‘0.03124’.

**Table S5. List of machine learning metrics for HMM-aided PDMP With different HMM thresholds. (Selected HMM thresholds for interventions are shown in the darker shade)**

| HMM  Thre-  Shold | True  Positive | Total  Positive | False  Positive | Total  Negative | Sensitivity | Specificity | Concordance Probability  (i.e., recall × specificity) | Accuracy | F1 Score |
| --- | --- | --- | --- | --- | --- | --- | --- | --- | --- |
| **0** | **4567.91** | **4567.91** | **425839.52** | **425839.52** | **1** | **0** | **0** | **0.01061299** | **0.02100308** |
| **0.1** | **9554.81** | **10445.48** | **329472.29** | **399859.64** | **0.91473154** | **0.17603014** | **0.16102032** | **0.19483588** | **0.05468131** |
| **0.2** | **9190.85** | **14834.8** | **2355.83** | **315098.89** | **0.61954661** | **0.99252352** | **0.61491458** | **0.97575337** | **0.69676531** |
| **0.3** | **8895.44** | **14642.23** | **994.39** | **314822.43** | **0.60751948** | **0.99684143** | **0.60560059** | **0.97953899** | **0.72520938** |
| **0.4** | **8629.62** | **14396.04** | **232.96** | **314387.12** | **0.59944401** | **0.999259** | **0.59899983** | **0.98175278** | **0.74205778** |
| **0.5** | **8416.59** | **14307.3** | **107.4** | **314790.2** | **0.58827242** | **0.99965882** | **0.58807171** | **0.98177406** | **0.73728554** |
| **0.6** | **5687.29** | **12120.58** | **63.73** | **314857.54** | **0.4692259** | **0.99979759** | **0.46913092** | **0.98013011** | **0.6364612** |
| **0.7** | **5816.65** | **12476.96** | **32.5** | **315085.16** | **0.46619128** | **0.99989685** | **0.4661432** | **0.97956781** | **0.63479375** |
| **0.8** | **4393.35** | **11508.36** | **18.97** | **315487.51** | **0.38175292** | **0.99993987** | **0.38172996** | **0.97818327** | **0.55190482** |
| **0.9** | **2989.35** | **10514.57** | **8.71** | **315671.97** | **0.28430549** | **0.99997241** | **0.28429765** | **0.976903** | **0.44245273** |
| **1** | **18.31** | **10202.11** | **0.21** | **316276.42** | **0.00179473** | **0.99999934** | **0.00179473** | **0.96880649** | **0.00358295** |

**Table S6. Results for single interventions: Percentage change from the baseline over five years**

| Policy | Medical overdoses (%) | Nonmedical overdoses (%) | Shift to street opioid use (%) | Legitimate opioid prescriptions (%) | Illegitimate opioid prescriptions (%) | Total filled opioid prescriptions (%) |
| --- | --- | --- | --- | --- | --- | --- |
| **Reduced prescription dose** |  |  |  |  |  |  |
| Reduction by 5% | -0.25% | -1.27% | -0.90% | -0.04% | -3.07% | -0.13% |
| Reduction by 10% | -3.12% | -5.29% | -0.52% | -0.12% | -2.95% | -0.21% |
| Reduction by 15% | -5.32% | -5.02% | -1.65% | +0.03% | -2.61% | -0.05% |
| Reduction by 20% | -8.60% | -9.45% | -3.62% | -0.04% | -4.57% | -0.18% |
| Reduction by 25% | -11.41% | -12.17% | -3.85% | -0.09% | -6.86% | -0.30% |
| **Reduced treatment duration** |  |  |  |  |  |  |
| Reduction by 5% | -2.21% | -6.83% | +2.67% | +1.23% | +1.22% | +1.23% |
| Reduction by 10% | -5.88% | -9.85% | +3.13% | +2.48% | +4.48% | +2.54% |
| Reduction by 15% | -7.59% | -14.33% | +6.01% | +3.80% | +4.85% | +3.83% |
| Reduction by 20% | -13.00% | -19.75% | +6.38% | +5.14% | +7.88% | +5.23% |
| Reduction by 25% | -14.47% | -27.13% | +8.24% | +6.63% | +10.52% | +6.75% |

**Table S7. Results for the simple PDMP intervention and HMM-aided PDMP interventions: Percentage change from the baseline over five years**

| Policy | Medical overdoses (%) | Non-medical overdoses (%) | Shift to street opioid use (%) | Legitimate opioid prescriptions (%) | Illegitimate opioid prescriptions (%) | Total filled opioid prescriptions (%) |
| --- | --- | --- | --- | --- | --- | --- |
| PDMP |  |  |  |  |  |  |
| Simple PDMP | +0.24% | -0.59% | +101.97% | -0.59% | -41.89% | -1.87% |
| **HMM-aided PDMP** |  |  |  |  |  |  |
| HMM threshold: 0.20 | +0.63% | -2.11% | +105.43% | -1.19% | -44.30% | -2.53% |
| HMM threshold: 0.30 | +0.03% | -3.52% | +98.42% | -0.94% | -43.88% | -1.99% |
| HMM threshold: 0.40 | +0.24% | -4.20% | +94.97% | -0.67% | -43.45% | -1.99% |
| HMM threshold: 0.50 | -0.12% | -1.84% | +91.35% | -0.60% | -42.71% | -1.90% |

**Table S8. Results of combinations of dual reductions in prescription doses and treatment duration: Percentage change from the baseline over five years.**

| Policy |  | Medical overdoses (%) | Non-medical overdoses (%) | Shifts to street opioid use (%) | Legitimate opioid prescriptions (%) | Illegitimate opioid prescriptions (%) | Total filled opioid prescriptions (%) |
| --- | --- | --- | --- | --- | --- | --- | --- |
| Reduced treatment duration by | Reduced prescription doses by |  |  |  |  |  |  |
| 5% | 5% | -3.95% | -7.72% | +1.81% | +1.15% | +0.54% | +1.14% |
| 5% | 10% | -4.71% | -9.74% | +0.71% | +1.13% | -1.86% | +1.04% |
| 5% | 15% | -8.20% | -13.08% | -0.10% | +1.18% | -1.87% | +1.08% |
| 5% | 20% | -11.29% | -10.97% | -2.80% | +1.16% | -2.74% | +1.04% |
| 5% | 25% | -13.99% | -15.15% | -1.92% | +1.10% | -4.60% | +0.93% |
| 10% | 5% | -7.57% | -13.44% | +4.28% | +2.49% | +4.07% | +2.53% |
| 10% | 10% | -8.90% | -14.67% | +3.42% | +2.46% | +1.72% | +2.44% |
| 10% | 15% | -12.47% | -16.01% | +0.29% | +2.37% | -0.98% | +2.26% |
| 10% | 20% | -13.38% | -18.69% | -0.43% | +2.43% | -1.73% | +2.30% |
| 10% | 25% | -15.51% | -22.30% | +0.38% | +2.46% | -0.82% | +2.36% |
| 15% | 5% | -10.97% | -18.35% | +3.82% | +3.69% | +2.91% | +3.67% |
| 15% | 10% | -11.04% | -20.00% | +2.95% | +3.77% | +3.52% | +3.76% |
| 15% | 15% | -13.65% | -20.35% | +3.46% | +3.80% | +2.99% | +3.77% |
| 15% | 20% | -16.24% | -25.25% | +4.16% | +3.81% | +3.88% | +3.82% |
| 15% | 25% | -19.50% | -26.29% | +0.37% | +3.75% | -0.29% | +3.62% |
| 20% | 5% | -14.45% | -24.75% | +7.36% | +5.13% | +8.03% | +5.22% |
| 20% | 10% | -14.83% | -25.64% | +5.27% | +5.19% | +6.51% | +5.23% |
| 20% | 15% | -16.65% | -27.57% | +4.71% | +5.22% | +5.88% | +5.24% |
| 20% | 20% | -20.27% | -28.47% | +2.88% | +5.14% | +2.82% | +5.06% |
| 20% | 25% | -21.06% | -32.24% | +2.80% | +5.07% | +1.95% | +4.98% |
| 25% | 5% | -16.16% | -26.57% | +7.90% | +6.60% | +11.43% | +6.75% |
| 25% | 10% | -20.30% | -30.95% | +6.81% | +6.52% | +8.10% | +6.57% |
| 25% | 15% | -19.94% | -31.83% | +5.03% | +6.56% | +6.01% | +6.54% |
| 25% | 20% | -23.51% | -33.76% | +4.61% | +6.55% | +4.99% | +6.50% |
| 25% | 25% | -25.73% | -38.03% | +4.10% | +6.52% | +5.15% | +6.47% |

**Table S9. Results of combinations of HMM-aided PDMP, lowering in prescription doses and lowering in treatment duration: Percentage change from the baseline over five years.**

| Policy | Medical overdoses (%) | Nonmedical overdoses (%) | Shifts to street opioid use (%) | Legitimate opioid prescriptions (%) | Illegitimate opioid prescriptions (%) | Total filled opioid prescriptions (%) |
| --- | --- | --- | --- | --- | --- | --- |
| HMM-aided PDMP threshold: 0.40 plus Lowering in prescription doses level 5 (25%) | -13.09% | -13.96% | +87.85% | -0.66% | -45.14% | -2.04% |
| HMM-aided PDMP threshold: 0.40 plus Lowering in treatment duration level 5 (25%) | -16.20% | -29.75% | +106.07% | +5.85% | -36.73% | +4.54% |
| HMM-aided PDMP threshold: 0.40 plus Lowering in treatment duration level 2 (10%) plus Lowering in prescription doses level 4 (20%) | -13.78% | -21.91% | +91.31% | +1.80% | -42.98% | +0.42% |

REFERENCES

1. Canadian Institute for Health Information. Opioid Prescribing in Canada: How Are Practices Changing?. Ottawa, ON: CIHI; 2019.

2. Miaskowski C, Dodd MJ, West C, Paul SM, Tripathy D, Koo P, Schumacher K. Lack of adherence with the analgesic regimen: a significant barrier to effective cancer pain management. *Journal of clinical oncology* (2001) 19:4275–4279.

3. Vowles KE, McEntee ML, Julnes PS, Frohe T, Ney JP, Van Der Goes DN. Rates of opioid misuse, abuse, and addiction in chronic pain: a systematic review and data synthesis. *Pain* (2015) 156:569–576.

4. Rosser BA, McCracken LM, Velleman SC, Boichat C, Eccleston C. Concerns about medication and medication adherence in patients with chronic pain recruited from general practice. *Pain* (2011) 152:1201–1205.

5. Lamplot JD, Premkumar A, James EW, Lawton CD, Pearle AD. Postoperative disposal of unused opioids: a systematic review. *HSS Journal®* (2021) 17:235–243.

6. Dunn KM, Saunders KW, Rutter CM, Banta-Green CJ, Merrill JO, Sullivan MD, Weisner CM, Silverberg MJ, Campbell CI, Psaty BM. Opioid prescriptions for chronic pain and overdose: a cohort study. *Annals of internal medicine* (2010) 152:85–92.

7. Durand Z, Nechuta S, Krishnaswami S, Hurwitz EL, McPheeters M. Prevalence and risk factors associated with long-term opioid use after injury among previously opioid-free workers. *JAMA network open* (2019) 2:e197222–e197222.

8. Shah A, Hayes CJ, Martin BC. Characteristics of initial prescription episodes and likelihood of long-term opioid use—United States, 2006–2015. *Morbidity and Mortality Weekly Report* (2017) 66:265.

9. Morgan MM, Christie MJ. Analysis of opioid efficacy, tolerance, addiction and dependence from cell culture to human. *British journal of pharmacology* (2011) 164:1322–1334.

10. Kosten TR, George TP. The neurobiology of opioid dependence: implications for treatment. *Science & practice perspectives* (2002) 1:13.

11. Sproule B. *Prescription monitoring programs in Canada: best practice and program review*. Canadian Centre on Substance Abuse Ottawa (2015).

12. Kaboré J-L, Choinière M, Dassieu L, Lacasse A, Pagé MG. Opioid Doctor Shopping: A Rare Phenomenon Among Patients with Chronic Non-Cancer Pain Followed in Tertiary Care Settings. *Journal of pain research* (2021)1855–1861.
